# Graph embedding and geometric deep learning relevance to network biology and structural chemistry

**DOI:** 10.3389/frai.2023.1256352

**Published:** 2023-11-16

**Authors:** Paola Lecca, Michela Lecca

**Affiliations:** ^1^Faculty of Engineering, Free University of Bozen-Bolzano, Bolzano, Italy; ^2^Fondazione Bruno Kessler, Digital Industry Center, Technologies of Vision, Trento, Italy

**Keywords:** deep learning, metric spaces, curved manifolds, latent network geometry, graph embedding, graph mining

## Abstract

Graphs are used as a model of complex relationships among data in biological science since the advent of systems biology in the early 2000. In particular, graph data analysis and graph data mining play an important role in biology interaction networks, where recent techniques of artificial intelligence, usually employed in other type of networks (e.g., social, citations, and trademark networks) aim to implement various data mining tasks including classification, clustering, recommendation, anomaly detection, and link prediction. The commitment and efforts of artificial intelligence research in network biology are motivated by the fact that machine learning techniques are often prohibitively computational demanding, low parallelizable, and ultimately inapplicable, since biological network of realistic size is a large system, which is characterised by a high density of interactions and often with a non-linear dynamics and a non-Euclidean latent geometry. Currently, graph embedding emerges as the new learning paradigm that shifts the tasks of building complex models for classification, clustering, and link prediction to learning an informative representation of the graph data in a vector space so that many graph mining and learning tasks can be more easily performed by employing efficient non-iterative traditional models (e.g., a linear support vector machine for the classification task). The great potential of graph embedding is the main reason of the flourishing of studies in this area and, in particular, the artificial intelligence learning techniques. In this mini review, we give a comprehensive summary of the main graph embedding algorithms in light of the recent burgeoning interest in geometric deep learning.

## 1 Introduction

Graph embedding is a mathematical procedure that transforms nodes, edges, and their features into vectors of a vector space (usually of a lower dimension) while trying to maximally preserve properties such as graph structure, vertex-to-vertex relationship, and other relevant information about graphs, subgraphs, and vertices. The uses of graph embedding are numerous, ranging from obtaining a representation of multidimensional data in a lower dimensional space for the purpose of more efficient manipulation and interpretation to the identification of the latent geometry of graphs. This second purpose is recently gaining ground in the analysis of biological networks, for which the geometry of the latent metric space explains organisational principles and dynamics (Krioukov et al., 2010; Krioukov, 2016; Bianconi and Rahmede, 2017; Alanis-Lobato et al., 2018; Papadopoulos and Flores, 2019; Boguñá et al., 2021; Lecca and Re, 2022).

Graph embedding can be implemented with artificial intelligence techniques, but it can also be used upstream of artificial intelligence techniques for learning and data analysis to enable easier and more efficient application of the techniques themselves. Graph embedding has a number of potential benefits. Embedding-based algorithms usually outperform their equivalents that employ the original networks in terms of speed. Additionally, downstream analysis frequently uses the learnt embeddings, either through a direct interpretation of the embedding space or through the use of machine learning methods specifically created for vectorial data (Nelson et al., 2019).

There are three types of embedding, namely, vertex embedding, edge/path embedding, and graph embedding proper. Vertex embedding is a mapping of graph vertices to vectors of a vector space (usually ℝ^*n*^), which approximately preserves properties such as distances between nodes. This type of embedding is commonly used to perform visualization or prediction on the vertex level or prediction of new connections based on vertex similarities. DeepWalk (Perozzi et al., 2014), node2vec (Grover and Leskovec, 2016), and SDNE (Wang et al., 2016) are three very well-known approaches to this type of embedding. Edge/path embedding is the mapping of graph edges to vectors of a vector space (usually of low dimension). This embedding is used to describe traversals across the graph and similarly to vertex embedding, targets edge prediction, reconstruction, and graph clustering (Wang et al., 2020). Finally, “graph embeddings” is a representation of the whole graph with a single vector (for example, Graph Readout operation in DGL-LifeSci, 2020). This type of embedding is used to make predictions on the graph level structure and compare or visualize the whole graphs, e.g., in studies of molecular structures where it is often necessary to compare chemical structures. In this review, however, we will not use the phrase “graph embedding” in this sense.

Graph embedding is critical to graph mining tasks such as classification, clustering, recommendation, anomaly detection, and link prediction. In the majority of applications, when the embedding is performed with the purpose to project the data in a lower dimensional space, the main reason for this lies in the possibility that the embedding offers to carry on these operations in simpler (in terms of handling and processing data structures and the usual operations on graphs) and more efficient manner. Network relationships in a graph of *V* nodes and *E* edges can only use a subset of mathematics, statistics, and machine learning, whereas vector spaces have a more diverse set of approaches (Pan et al., 2020). Graph embedding consists of calculating the coordinates of its nodes in a vector space so that the properties of the graph, such as, for example, the node content and the distances between nodes, are preserved within a certain error, which is desired to be small. The adjacency matrix is the most common numerical representation of a graph. It is a |*V*| × |*V*| matrix, where |*V*| is the number of graph nodes. In the matrix, each column and row represents a node, and non-zero values indicate that two nodes are connected. It is nearly impossible to use an adjacency matrix as a feature space for large graphs. Consider that a graph with 10^6^ nodes is represented by a 10^6^×10^6^ adjacency matrix. Since node properties are packed into a vector by embedding rather than the adjacency matrix, they are more useful. Additionally, compared with equivalent procedures on graphs, vector operations are easier and quicker. In this regard, on the basis of a certainty now shared by many scholars form different disciplines and in different contexts of application [e.g., Cao et al., 2016b; Goyal and Ferrara, 2018; Pan et al., 2020; Yang et al., 2020; Amara et al., 2021; Etaiwi and Awajan, 2023], Zhang et al. (2020) talk about a paradigm shift and explain that this new learning paradigm has shifted the tasks of seeking complex models for classification, clustering, and link prediction “to learning a compact and informative representation for the graph data” so that many graph mining tasks can be easily performed by employing simple traditional models (e.g., a linear Support Vector Machine for the classification task). Furthermore, because of how different they can be in terms of scale, specificity, and topic, graph embedding can be challenging. A social network could be depicted by a large, dense, dynamic graph as opposed to a small, sparse, and static representation of a molecule (Xiong et al., 2019; David et al., 2020). In the end, this makes it challenging to identify an optimal embedding technique of general validity and applicability. It is, therefore, necessary to focus on the analysis and review of specific application domains. In the following sections, we will describe and review the current graph embedding methods commoly used in systems biology.

## 2 Graph embedding algorithms

The three types of graph embedding algorithms are (i) probability models, (ii) algorithms based on matrix factorization, and (iii) algorithms based on deep learning. For a comparative review of preservation extent of current graph embedding methods, we refer the reader to the study by Goyal and Ferrara (2018), Mohan and Pramod (2019), Vaudaine et al. (2020), and Xu (2021). In addition to classification according to the mathematical and/or algorithmic methodology adopted, graph embedding approaches can also be classified according to the type of input they process.

**Probabilistic embedding:** Probabilistic embedding methods predict the distribution of embeddings, as opposed to deterministic embedding methods, which map the input to a single embedding vector. Probabilistic embeddings have the following benefits over deterministic methods: (i) probabilistic losses can stabilise training on noisy data; (ii) predicted uncertainty can be used to assess the quality of the data and identify out-of-domain data (see Karpukhin et al., 2022 for a more detailed discussion); (iii) tasks involving rejection and categorization may benefit from confidence (Chang et al., 2020; Mena et al., 2020; Karpukhin et al., 2022).

By extracting various patterns from the network, probabilistic models such as DeepWalk (Perozzi et al., 2014), node2vec (Grover and Leskovec, 2016), and LINE (Tang et al., 2015c) try to learn graph embedding. DeepWalk learns the node embedding from a set of random walks. Node2vec creates random walk sequences for network encoding by combining breadth-first sampling (BFS) and depth-first sampling (DFS) techniques. LINE handles massive information networks while maintaining both first-order and second-order proximities. Other random walk variations include DDRW [Discriminative Deep Random Walk (Li et al., 2016a)] and HARP (Hierarchical Representation Learning Approach, Zhao et al., 2023). Global structural equivalence, neighbourhood connectivities at the local level, and other different order proximities are among the recorded patterns or walks. These graph embedding algorithms outperform traditional approaches such as Spectral Clustering (von Luxburg, 2007) and are scalable to large networks (Pan et al., 2020).

**Matrix factorization-based embedding:** The adjacency matrix *A*∈ℝ^*m*×*n*^ is factorized by two matrices, namely, *U*∈ℝ^*m*×*d*^ and *V*∈ℝ^*n*×*d*^ (to be learnt), where *d* is the dimension of the embedding space. When compared with learning the entire matrix, matrix factorization usually produces a more concise representation. The embedding matrices *U* and *V* have *O*((*m*+*n*)*d*) entries, whereas the full matrix has *O*(*mn*) entries. The embedding dimension *d* in these matrices is usually much smaller than *m* and *n*. The embeddings are learned such that the *UV*^*T*^ well approximates the matrix *A*. The entry (*i, j*) of *U*, *V*^*T*^ is the inner product 〈*U*_*i*_, *V*_*j*_〉, which we want to be close to *A*_*ij*_.

Examples of factorisation-based embedding algorithms are GraRep (Graph Representations with Global Structural Information, Cao et al., 2015), HOPE (High-Order Proximity preserved Embedding, Ou et al., 2016), and M-NMF (Modularized Nonnegative Matrix Factorization, Yan and Chang, 2019), which then factorise the adjacency matrix to implement the embedding. GraRep, as well as NEU [Neural Network Embeddings (Yang et al., 2017)], and AROPE (Arbitrary-Order Proximity Preserved Network Embedding, Zhang et al., 2018) all capture the higher order approximation except the first-order and the second-order similarities. HOPE preserves asymmetric transitivity by approximating high-order proximity for improved performance in graph topological information capture and reconstruction from partially observed graphs. Among the graph embedding methods based on matrix factorisation, we also mention the recent study by Liu et al. (2020), who propose SMNMF, a Semisupervised Modularised Non-negative Matrix Embedding model. Liu et al. point out that the existing network representation learning algorithms are mostly unsupervised models, and that the pairwise constraint information, which represents community membership, is not effectively used to obtain node embedding results that are more consistent with previous knowledge. Their method, SMNMF, offers a network embedding procedure while preserving the community structure; the pairwise constraint (must-link and cannot-link) information is effectively fused with the network's adjacency matrix and node similarity matrix, making the node representations learned by the model more interpretable.

**Deep learning-based embedding:** Neural network embeddings are learned low-dimensional vector representations of discrete variables. The network's parameters, or weights, are formed by the embeddings and are adjusted to reduce task loss. The resulting embedded vectors indicate categories where related categories are closer to one another in relation to the task.

Pan et al. (2020) report that many probabilistic algorithms, such as DeepWalk (Perozzi et al., 2014), LINE (Large-scale Information Network Embedding) (Tang et al., 2015c), and node2vec (Grover and Leskovec, 2016), have lately been shown to be equivalent to matrix factorisation methods, and Qiu et al. (2018) have proposed a unified matrix factorisation method called NetMF (Network Embedding as Matrix Factorization) for graph embedding. The methods are unify DeepWalk, LINE, PTE [Predictive Text Embedding (Tang et al., 2015a)], and node2vec.

Deep learning techniques based on autoencoders are also being researched (Zhu et al., 2019; Hasibi and Michoel, 2021; Xu, 2021; Wang et al., 2022). SDNE (Structural Deep Network Embedding) (Wang et al., 2016) and DNGR (Deep Neural Networks for Graph Representations) (Cao et al., 2016a) use deep autoencoders (Baldi, 2012) to preserve the graph proximities and model the positive pointwise mutual information (PPMI). To learn representation for graph clustering, the MGAE (Marginalized Graph Autoencoder) algorithm relies on a marginalised single layer autoencoder (Wang et al., 2017). For signed network embedding with a stacked auto-encoder framework, the DNE-SBP (Deep Network Embedding with Structural Balance Preservation) model is proposed (Shen and Chung, 2020).

**Input-based classification of embedding algorithm:** According to Pan et al. (2020), the embedding algorithms for graph mining can be further classified into *topological network embedding* approaches and *content enhanced network embedding* methods. The former take as an input only the information concerning the topological structure of the graph, and their learning objective is to preserve it maximally. The latter take as an input the node and process both topological information and content features.

DeepWalk, node2vec, LINE [Large-scale Information Network Embedding (Tang et al., 2015b)], HARP (hierarchical representation learning approach, Chen et al., 2018), DDRW (Discriminative Deep Random Walk, Li et al., 2016b), and Walklets (Perozzi et al., 2016) are *topological network embedding* approaches. DeepWalk can be interpreted as a factorisation method, and Yang et al. introduced TADW. They also suggested extending DeepWalk to investigate node features. Many other embedding algorithms process both topological and node content information, e.g., TriDNR [Tri-Party Deep Network Representation (Pan et al., 2016)], UPPSINE [User Profile Preserving Social Network Embedding (Zhang et al., 2017)], ASNE [Attributed Social Network Embedding (Liao et al., 2018)], LANE [Label informed Attributed Network Embedding (Huang et al., 2017)], DANE [Domain Adaptive Network Embedding (Song et al., 2022)], and BANE [Binarized attributed network embedding (Yang et al., 2018)].

TriDNR uses a tri-party neural network architecture to record information about structure, node content, and labels. UPPSNE uses an approximated kernel mapping scheme to take the advantage of user profile characteristics in order to maximise the learning that users are embedding into social networks. In essence, a kernel approximation mapping is learning an estimated subspace in the high-dimensional feature vector space that is induced and defined by the kernel function (Francis and Raimond, 2020). For attributed social networks, ASNE trains a neural network model to incorporate both structural proximity and attribute proximity, and LANE integrates label information into the optimization process to learn a better embedding. This is, in general, a difficult task because of the possibility of noisy and incomplete label information. Additionally, labels, geometrical structure, and node properties are totally distinct from one another. Finally, recently, BANE was suggested to learn binarised embedding for an attributed graph, which could improve efficiency for later graph analytical tasks.

Most graph embedding methods have been developed principally for indirect graphs, since (i) information about the direction of the arcs leads to a non-symmetrical weighted adjacency matrix (the factorisation of which is more complex), and (ii) the metric space that can possibly describe the latent geometry of a directed graph must have characteristics such that it can store information about directionality. For this reason, we devote the next section to those embedding techniques specialised for directed graphs. For the sake of completeness, we will then discuss in the following sections the embedding methods for three other categories of graphs (bipartite, temporal, and multi-label), which, with their topological peculiarities, are useful in describing numerous real-world networks. We refer the reader to the study by Kamiński et al. (2019), Vaudaine et al. (2020), Zhang et al. (2021c) for a comparison of different embedding methods.

### 2.1 Embedding of directed graphs

For undirected graphs, the weighted adjacency matrix (alias *affinity matrix*) is symmetrical, and thus, for each pair of nodes, the weight of the arcs joining them and/or the dissimilarity or distance between them are uniquely defined, since they have no dependence on the direction of the arc. The symmetry of the affinity matrix greatly simplifies the operations and computational procedures of the embedding methods. However, there is a significant amount of intrinsically asymmetric graph data, such as, for example, social networks, alignment scores between biological sequences, and citation data. As reported by Perrault-joncas and Meila (2011), a common strategy for this type of data is not to use as input for embedding procedures, the asymmetric affinity matrix *W*, but the matrices obtained from it as *W*+*W*^*T*^ or *W*^*T*^*W*. In fact, suppose that


$$\begin{array}{l} W=\left(\begin{array}{cc} a & b\\ c & d \end{array}\right) \end{array}$$

where *a*≠*d* and *b*≠*c*. Then,


$$\begin{array}{l} W+W^{T}=\left(\begin{array}{cc} 2a & b+c\\ b+c & 2d \end{array}\right) \end{array}$$

and


$$\begin{array}{l} W^{T}W=\left(\begin{array}{cc} a^{2}+b^{2} & ab+cd\\ b+c & b^{2}+c^{2} \end{array}\right). \end{array}$$

are both symmetric matrices.

Already in the early 2000s, other approaches have been proposed to directly deal with the asymmetry of the affinity matrix (Zhou et al., 2004, 2005; Meilă and Pentney, 2007) or define directed Laplacian operators (Chung, 2006).

Interest in and the need to develop efficient *ad hoc* methods for embedding directed graphs have re-emerged very recently, and the attention of the community has focused mainly on embedding in non-Euclidean spaces. Indeed, it is becoming more and more apparent that Euclidean geometry cannot easily encode complex interactions on big graphs and is not flexible to handle edge directionality. Of interest, as being more functional for geometric deep learning, we mention the study by Sim et al. (2021). The authors of this study demonstrate that directed graphs can be efficiently represented by an embedding model that combines three elements, namely, a non-trivial global topology (directed graphs, eventually containing cycles), a pseudo-Riemannian metric structure, and an original likelihood function that explicitly takes into account a preferred direction in embedding space.

Pseudo-Riemannian manifolds are Riemannian manifold generalisations that relax the requirement of the non-degenerate metric tensor's positive definiteness. As comprehensively described in the study by Law and Lucas (2023), there are two categories in the machine learning literature on pseudo-Riemannian manifolds. The first category does not consider whether the manifold is time-oriented or not; instead, it concentrates on how to optimise a given function whose domain is a pseudo-Riemannian manifold (Law and Stam, 2020; Law, 2021). The second category makes the use of how general relativity interprets a particular family of pseudo-Riemannian manifolds known as “spacetime” (Clough and Evans, 2017; Sim et al., 2021). Spacetimes are actually linked time-oriented Lorentz manifolds. They inherently have a causal structure that shows whether or not there is a causal chain connecting occurrences at different positions on the manifold. In directed graphs, each node is an event, and the existence of a directed arc between two nodes depends on the causal characteristics of the curves connecting them (Bombelli et al., 1987). This causal structure has been used to depict these directed networks.

Sim et al. (2021), on the other hand, use three different spacetime types and suggest an *ad hoc* method introducing a time coordinate difference function, whose sign is then used to determine the orientation of edges. This has been an interesting and innovative approach that suggested further research and advancement as indicated by Law and Lucas (2023). In that study, Law et al. observe, regarding the study by Sim el al., that when the manifold is non-chronological and does not generalise to all spacetimes, the sign of such a function, for example, alternates periodically and is not always relevant. Additionally, when a geodesic cannot connect two points, their distance function remains constant.

In this brief subsection, we have emphasized embedding methods of directed graphs that adopt spacetime representations. Indeed, the wealth of information contained in spatiotemporal structures could inspire the design of a neural network that can learn most of it and can thus become as accurate a tool for geometric deep learning as possible. For the sake of completeness, however, we mention some other recent studies that propose other methods for embedding directed graphs, such as ShortWalk algorithm by Zhao et al. (2021), MagNet by Zhang et al. (2021a), and the study of Khosla et al. (2020). Less recent is the study of Chen et al. (2007) that proposed an approach taking into account the link structure of graphs to embed the vertices of a directed graph into a vector space.

The idea of ShortWalk is that long random walks may become stuck or stopped in a directed graph, producing embeddings of poor quality. To enhance the directed graph network embeddings, ShortWalk generates shorter traces by doing brief random walks that restart more frequently. MagNet uses a graph neural network for modelling directed graphs, exploiting a complex Hermitian matrix which encodes undirected geometric structure as the magnitude of the matrix entries and the direction of edges in their phase. Khosla et al. (2020) created role-specific vertex neighbourhoods and trained node embeddings in their associated source/target roles using *alternating random walk technique*, fully using the semantics of directed graphs. Alternating walk technique draws inspiration from SALSA (Lempel and Moran, 2001), a stochastic variant of the HITS (Kleinberg, 1999) algorithm that also recognises hubs and authorities as two different categories of significant nodes in a directed network. The pathways produced by alternating random walks between hubs (source nodes) and authorities (target nodes), sampling both nearby hubs and authorities in relation to an input node.

In the study by Chen et al. (2007), the main goal of the authors was to keep vertices on the locality property of a directed graph in the embedded space. To assess this locality quality, they combined the transition probability with the stationary distribution of Markov random walks. It turns out that they obtained an ideal vector space embedding that maintains the local affinity that is inherent in the directed graph by utilising random walks to explore the directed links of the graph.

Finally, it is worth mentioning that most of the directed graph embedding techniques mentioned in this section are also appropriate for embedding bipartite graphs. A comprehensive survey on bipartite graph embedding can be found in the study by Giamphy et al. (2023), and a short review is also presented in the next sub-section.

### 2.2 Embedding of bipartite graphs

Since bipartite graphs are frequently utilised in many different application domains [many of them in biology and medicine (Pavlopoulos et al., 2018; Ma et al., 2023) and drug discovery an repurposing (Zheng et al., 2018; Manoochehri and Nourani, 2020; Hostallero et al., 2022; Yu et al., 2022)], bipartite graph embedding has recently received a lot of interest. The majority of earlier techniques, which use random walk- or reconstruction-based objectives, are usually successful at learning local graph topologies. However, the general characteristics of the bipartite network, such as the long-range dependencies of heterogeneous nodes and the community structures of homogeneous nodes, are not effectively retained. To circumvent these constraints, Cao et al. (2021) developed BiGI, a bipartite graph embedding that captures such global features by introducing a novel local-global infomax objective. BiGI generates a global representation initially, which is made up of two prototype representations. The suggested subgraph-level attention method is then used by BiGI to encode sampled edges as local representations. BiGI makes nodes in a bipartite graph globally relevant by maximising mutual information between local and global representations.

Also the Yang et al. (2022b) proposal for efficient embedding of bipartite graphs of big size is of interest. Yang et al. pointed out that existing solutions are rarely scalable to vast bipartite graphs, and they frequently produce subpar results. The study by Yang et al. (2022b) introduced Generic Bipartite Network Embedding (GEBE), a general bipartite network embedding (BNE) framework, with four core algorithmic designs that achieves state-of-the-art performance on very large bipartite graphs. First, GEBE provides two generic measures that may be instantiated using three popular probability distributions, such as Poisson, Geometric, and Uniform distributions, to capture multi-hop similarity (MHS)/ multi-hop proximity (MHP) between homogeneous/heterogeneous nodes. Second, GEBE develops a unified BNE goal to preserve the two measurements of all feasible node pairs. Third, GEBE includes a number of efficiency strategies for obtaining high-quality embeddings on large graphs. Finally, according to the study by Yang et al. (2022b), GEBE performs best when MHS and MHP are instantiated using a Poisson distribution, therefore they continued to build GEBEp based on Poisson-instantiated MHS and MHP with challenging efficiency improvements.

### 2.3 Embedding of temporal graphs

A network whose links are not continuously active is referred to as a temporal network or a time-varying network (Holme and Saramäki, 2012). Each connection includes its active time as well as any further details that may apply, including its weight. Traditional network embedding methods are created for static structures, frequently taken into account nodes, and face significant difficulties when the network is changing over time. Various methods for embedding temporal networks have been proposed in the last years, e.g., Stwalk (Pandhre et al., 2018), tNodeEmbed (Singer et al., 2019), Online Node2Vec (Béres et al., 2019), and the Dynamic Bayesian Knowledge Graphs Embedding model of Liao et al. (2021). In many methods of temporal network embedding, the traditional representation of the temporal network is often modified, whether it takes the form of a list of events, a tensor [as in the method by Gauvin et al. (2014)], or a supra-adjacency matrix [as in DyANE algorithm by Sato et al. (2021)]. Each of these approaches, such as DyANE, Online Node2Vec, STWalk, and tNodeEmbed, share the common goal of resolving the node embedding issue by locally sampling the temporal–structural neighbourhood of nodes to produce contexts, which they then feed into a Skip-Gram learning architecture adapted from the text representation literature. They have been used in chemical and biological research studies, e.g. Kim et al. (2018), Öztürk et al. (2020), Fang et al. (2021), and Gharavi et al. (2021) and in biomedical language processing (Zhang et al., 2019b).

Regarding these studies, Torricelli et al. (2020) highlighted that as a workaround these methods construct a series of correlated/updated embeddings of network snapshots that take into account the network's recent past. Torricelli et al. stated that the main drawback of these approaches is that it might be challenging to control a large number of hyper-parameters for the sampling random walk process and the embedding itself. The embedding of nodes may, however, fail to capture the dynamic shifts in temporal interconnections. The performance of the prediction might be severely hindered by simply considering past and current interactions in the embedding, whereas bringing future occurrences into account can greatly enhance this task. Torricelli et al. (2020) developed weg2vec to overcome these limitations. Weg2vec learns a low-dimensional representation of a temporal network based on the temporal and structural similarity of occurrences. A higher order static representation of temporal networks, sampled locally by weg2vec, codes the intricate patterns that define the structure and dynamics of real-world networks. This method of unsupervised representation learning can concurrently take into account an event's past and future contexts. It samples without the use of dynamical processes, making it controllable by a few hyper-parameters. It finds similarities between various events or nodes that may be active at various periods but have an impact on a group of nodes that are similar in future.

Finally, the studies by Yang et al. (2022a) proposed a hyperbolic temporal graph network on the Poincaré ball model of hyperbolic space; the temporal network embedding using graph attention network by Mohan and Pramod (2021); the embedding based on a variational autoencoder able to capture the evolution of temporal networks by Jiao et al. (2022), ConMNCI by Liu et al. (2022) that inductively mines local and communal influences. The authors of ConMNCI suggested an aggregator function that combines local and global influences to produce node embeddings at any time and presented the concept of continuous learning to strengthen inductive learning; the continuous-time dynamic network embeddings by Nguyen et al. (2018), the causal anonymous walk representations for temporal network embedding by Makarov et al. (2022); and TempNodeEmb by Abbas et al. (2023). To extract node orientation using angle approach, the methodology by Abbas et al. considered a three-layer graph neural network at each time step, taking advantage of the networks' ability to evolve.

Finally, we mention a study aimed at the possibility of using embedding methodologies in practice for networks of degree sizes, i.e., the proposal for parallelising temporal network embedding procedure by Xie et al. (2022).

### 2.4 Embedding of multi-label graphs

The graph convolution network (GCN) is a widely-used method to embed multi-label graphs (Ye and Wang, 2022). However, Gao et al. (2019), pointed out that for multi-label learning problems, the supervision component of GCN just minimises the cross-entropy loss between the last layer outputs and the ground-truth label distribution, which often misses important information such as label correlations and prevents obtaining high performance. In this study, the authors proposed ML-GCN, a semi-supervised learning approach for Multi-Label classification based on GCN. ML-GCN first makes the use of a GCN before including the node attributes and graph topological data. Then, a label matrix is generated at random, with each row (or label vector) denoting a different type of label. Before the most recent convolution operation performed by GCN, the label vector's dimension was the same as the node vector's. In other words, every label and node is contained within a constant vector space. The label vectors and node vectors are finally concatenated during the ML-GCN model training to serve as the inputs of the relaxed Skip-Gram model to identify the node-label correlation and the label-label correlation.

Another study by Shi et al. (2020), to learn feature representation for networked multi-label instances, presented an interesting multi-label network embedding (MLNE) method. The key to MLNE learning is to combine node topological structures, node content, and multi-label correlations. To couple information for successful learning, the authors developed a two-layer network embedding approach. To capture higher order label correlations, the authors employed labels to construct a high-level label-label network on top of a low-level node-node network, with the label network interacting with the node network *via* multi-labelling interactions. Latent label-specific characteristics from a high-level label network with well-captured high-order correlations between labels are used to improve the low-level node-node network. In MLNE, both node and label representations are forced to be optimised in the same low-dimensional latent space to enable multi-label informed network embedding.

Recent successful applications of the embedding for multi-label classification in medical domain is the medical term semantic type classification (Yue et al., 2019) and the knowledge graph embedding to profile transcription factors and their target genes interaction (Wu et al., 2023).

### 2.5 On the complexity of graph embedding

To conclude this section on graph embedding, we would like to provide some information about the complexity of the embedding.

Due to the high dimensionality and heterogeneity of real-world size networks, classical adjacency matrix-based techniques suffer from high computational costs and prohibitive memory needs. The computational complexity of adjacency matrix approaches is at best *O*(*n*^2^) (where *n* is the number of nods of the graph).

Random walk-based methods, on the other hand, proved as more efficient in terms of both space and time requirements than both matrix factorisation and BFS/DFS-based methods. For example in node2vec (Grover and Leskovec, 2016), the space complexity for storing each node's closest neighbours is *O*(*e*) (where *e* is the number of edges). The links between each node's neighbours need to be stored for 2nd order random walks, incurring a space complexity of *O*(*d*^2^*n*) (here *d* is the average degree of the graph, and *n* the number of nodes). Random walks have a temporal complexity advantage over conventional search-based sampling techniques too. Random walks, in particular, offer a straightforward way to boost the effective sampling rate by reusing samples across several source nodes by imposing network connectedness in the sample production process.

The increasing of methods for graph embedding obviously prevents us from making generalisations or categorisations regarding the complexity of the various methods. Here, we wanted to report the information in this regard that is confirmed in the literature. At the moment of writing, and to the best of our knowledge, systematic studies on estimating the complexity of each proposed method are very limited, if any, part of the literature on the subject.

To conclude, we mention the studies of Archdeacon (1990) and Chen et al. (1993) on the complexity of graph embedding procedures. It is well-known that embedding a graph G into a surface of minimum genus γ_min_(*G*) is NP-hard whereas embedding a graph G into a surface of maximum genus γ_max_(*G*) can be done in polynomial time. Chen et al. demonstrated that the problem of embedding a graph *G* with *n* vertices into a surface with genus at most γ_min_(*G*)+*f*(*n*) remains NP-hard, but there is a linear time algorithm that approximates the minimum genus embedding either within a constant ratio or within a difference *O*(*n*) for any function *f*(*n*^ϵ^) = *O*(*n*) where 0 ≤ ϵ ≤ 1.

## 3 Geometric deep learning

A technique that takes into account a large class of machine learning issues from the perspectives of symmetry and invariance is known as *geometric deep learning* (Bronstein et al., 2017, 2021).

An example provided by R. East in East (2023) intuitively and effectively explains the concepts of symmetry and invariance in machine learning. R. East imagines that we are given a learning task and the data we are using to learn from has an underlying symmetry and takes as an example of the game of Noughts and Crosses. East points out that if we win, we would have won if the board had been rotated or flipped along any of the lines of symmetry. Moreover, we have two options if we want to train an algorithm to predict the result of these games: we can either disregard this symmetry or include it in some way. The benefit of focusing on the symmetry is that it recognises various board configurations as “the same thing” as far as the symmetry is concerned. As a result says East, we can immediately lower the quantity of data, and our algorithm must sort through by reducing the size of our parameter space. Our results are also naturally encouraged to be more generalisable by the fact that the learning model must encode a symmetry that truly exists in the system we are seeking to portray.

If we ignore the possibility that the majority of tasks of interest are not generic and come with necessary predefined regularities arising from the latent geometry of the physical system under investigation, learning generic functions in a given number of dimensions is a cursed estimation problem. Through unified geometrical concepts that may be used in a wide range of applications, geometric deep learning aims to exploit these regularities. Indeed, a powerful and time-tested solution to the dimensionality curse, and the foundation of most physical theories, is to take advantage of the system's recognised symmetries. Deep learning systems are not different. For instance, researchers have modified neural networks to exploit the geometry, resulting from physical measurements, such as grids in images, sequences in time-series, or position and momentum in molecules, and their associated symmetries, such as translation or rotation. The knowledge about the geometry of the physical systems is often referred to as *geometric*. These priors include the concepts of symmetry and invariance, stability, and multiscale visualisations. In summary, we can define geometric deep learning as a machine learning technique in which the machine, instructed by geometric priors, learns from complicated data represented by graphs or multidimensional points, often in non-Euclidean domains. Tong in an online study (Tong, 2019) uses a very effective example to explain the difference between deep learning and geometric deep learning. He says that “The difference between traditional deep learning and geometric deep learning can be illustrated by imagining the accuracy between scanning an image of a person versus scanning the surface of the person themselves”.

**Figure 1:**
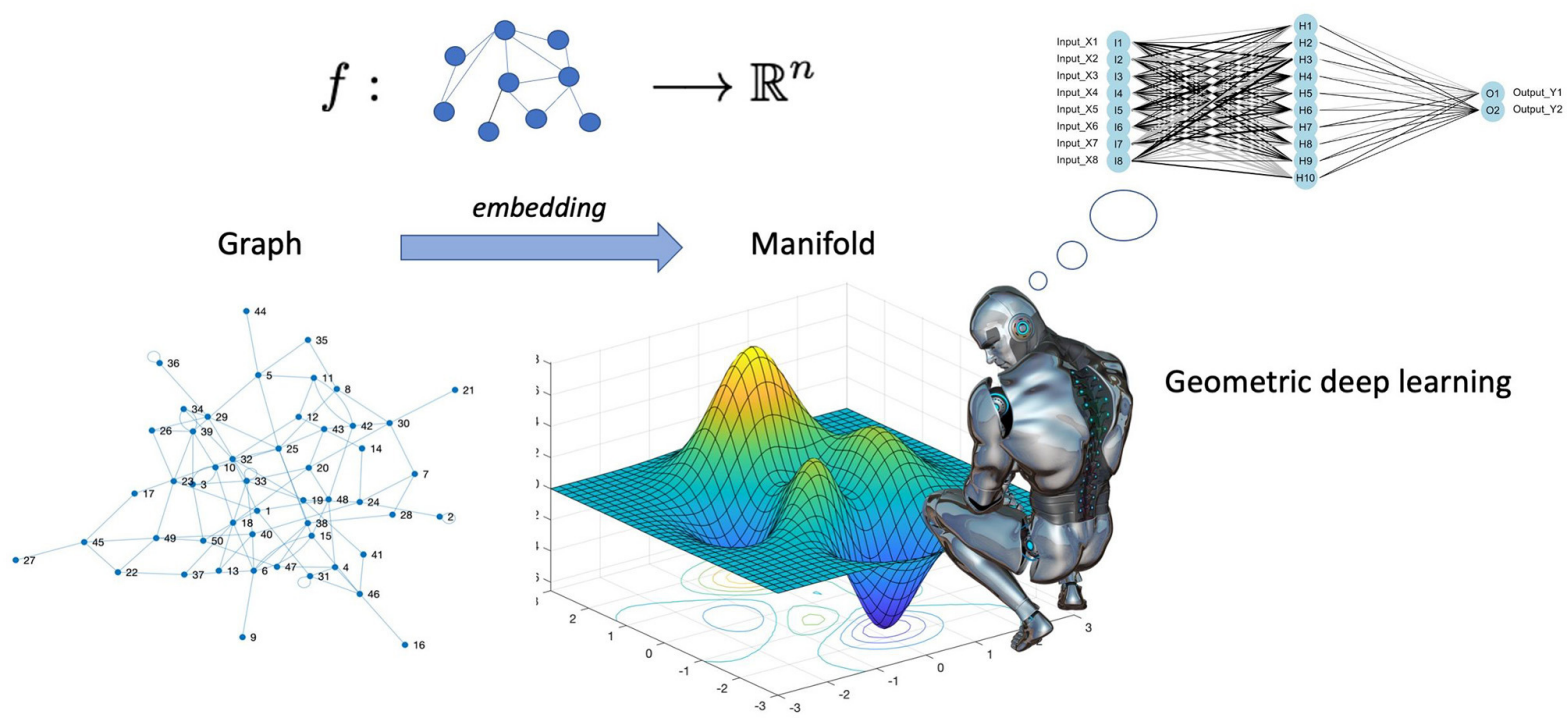
Geometric deep learning involves encoding a geometric understanding of the graph, e.g., the knowledge of nodes' coordinates, and their distances in the latent metric space of the graph, and creates neural networks that can learn from this geometry who, in the majority of the real networks, is not Euclidean. In spite of the recent flourishing of the application of graph neural networks to biological sciences, a plethora of successful case studies populate the current literature [see for example the review by Zhang et al. (2021b)].

**Figure 2:**
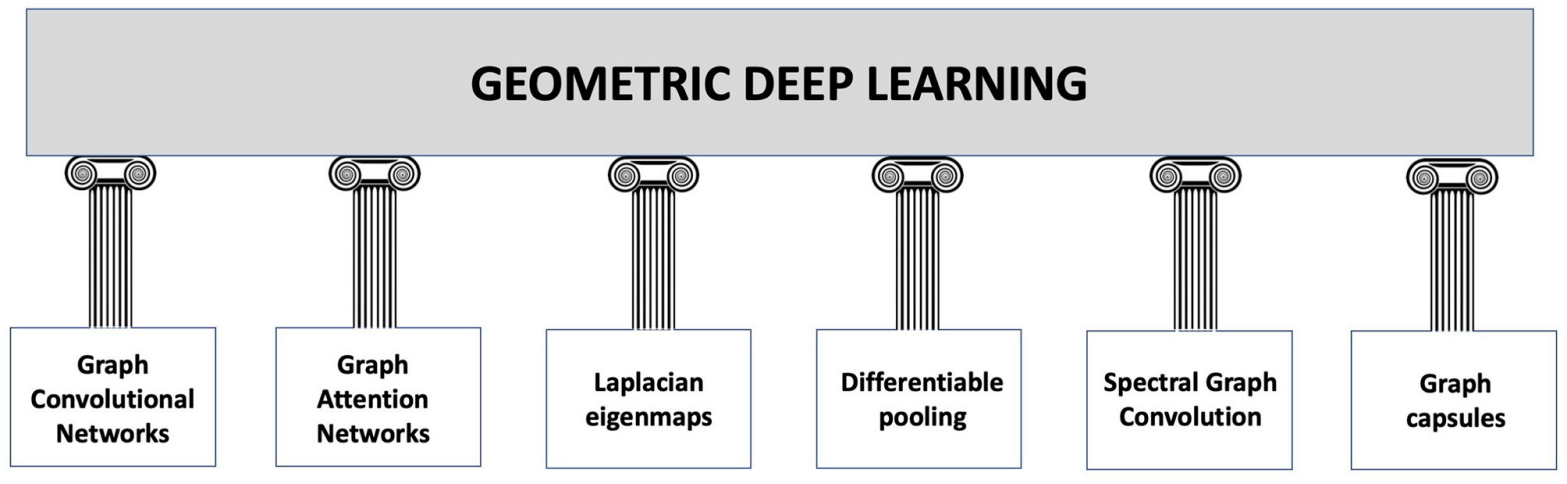
Building blocks of geometric deep learning according to the study by Sivakumar (2023). **Graph Convolutional Networks (GCNs)** are a particular type of neural network that may be used to categorise and extract characteristics from data that are graph-structured. The convolution technique, which merges data from nearby nodes in a graph, serves as their foundation. **Graph Attention Networks (GATs)** are a subset of GCNs that employ attention mechanisms to assess the relative relevance of various nodes in a graph. This enables GATs to process the graph while concentrating on the most important nodes. **Laplacian eigenmaps:** By mapping graph-structured data to a lower-dimensional space, a method known as Laplacian eigenmaps is utilised to reduce the dimensionality of the data. This is accomplished by identifying the eigenvectors of the graph's Laplacian matrix. **Differentiable Pooling:** This technique enables for the handling of variable-size graph-structured data by dynamically adjusting the number of nodes in a graph during the training phase (references in Ying et al., 2018; Li et al., 2021a). **Spectral Graph Convolution** is a method for handling graph-structured data that are based on the eigenvectors of the graph Laplacian. It enables the convolving of a signal on the graph by the use of a Fourier space filter (some references in Zhang et al., 2019a; Salim and Sumitra, 2022). **Graph capsules:** It is a technique that makes use of a capsule network design to enhance the functionality of graph-based models (Misra, 2019). A certain class of neural network called capsules may simulate relationships between things.

Cao et al. (2020) go even further in explaining the difference between deep learning and geometric deep learning as follows. In several machine learning applications, such as object identification, image classification, speech recognition, and machine translation, the deep learning technologies, for instance, the convolutional neural networks (CNN)s, have produced exceptional results. Convolutional neural networks, in particular, leverage the fundamental statistical properties of data, such as local stationarity and multi-scale component structure, to capture more detailed local features and information than classic neural networks. Although deep learning technology is particularly effective in processing conventional signals such as images, sound, video, or text, current deep learning research still primarily concentrates on the aforementioned data that are defined in the Euclidean domain, i.e., grid-like data. However, as higher data scales and more powerful GPU computing capabilities emerge, people are becoming more and more interested in processing data in non-Euclidean domains. Here, we point out that graphs are endowed with latent geometry. By virtue of this, nodes and edges have spatial features, such as coordinates of nodes and directions along edges, which are geometric relationships (including distance, direction, and angle) among the entities for a graph neural network to learn from (see Figure 1 which suggest first to embed the graph in the least distorting manifold and then to apply geometric deep learning). In the previous section, we saw that embedding procedures give an indication which manifold best describes the latent metric space of the graph and calculates the nodes' coordinates and distances in that manifold. Knowledge of the manifold describing the graph is thus an additional information that enriches the input for a neural network. However, manifold learning poses difficult challenges to traditional deep learning. When describing geometric shapes, such as the surfaces of objects, the manifold data are typically utilised. Cao et al. (2020) point out that it is difficult to identify the underlying pattern in these geometric data because they may be randomly dispersed and unevenly structured. Non-Euclidean data typically have an exceptionally vast scale, which is another problem. A molecular network, for instance, may include hundreds of millions of nodes. It seems unlikely that typical deep learning technology can be used in this situation to perform analysis and prediction. Indeed, geometric deep learning is much more articulate than traditional deep learning and involves fundamental building elements, as presented in Figure 2. The convolution technique, which merges data from nearby nodes in a graph, serves as their foundation of each of these elements.

### 3.1 Applications

In Table 1, we summarize the main categories of the geometric deep learning and the current main applications of it. Various applications of geometric deep learning to the study of biological systems, especially at the level of dynamic interactome, are beginning to emerge, and we will make special mention of these in this review.

**Table 1:** Categories and applications of geometric deep learning.

**Geometric deep learning**	
**Categories**	**Applications**
**Graph Neural Networks (GNNs)**. Examples of GNNs include Graph Convolutional Networks (GCNs) (Zhang et al., 2019a) and Graph Attention Networks (GATs) (Veličković et al., 2018; Hong et al., 2020; Chen et al., 2022).	Image classification, object detection and segmentation, natural language processing, structural chemistry, molecule solubility, network node classification, and recommendation systems.
**Manifold-valued Neural Networks (MVNs)**. Examples of MVNs include Spherical CNNs (Li et al., 2021b; Su and Grauman, 2021; Scott et al., 2022), PointNet (Charles et al., 2017), and ManifoldNet (Chakraborty et al., 2022).	Image classification, object detection and segmentation, natural language processing, and recommendation systems.
**Topology and Shape Analysis**. Case studies and surveys in the study by Hensel et al. (2021) and Magai and Ayzenberg (2022).	Analysis of properties of data, such as connectivity, homology, and curvature, is used in fields such as computer vision, medical imaging, and computational biology.

When it comes to operating on graph-structured and manifold-valued data, geometric deep learning can be roughly divided into Graph Neural Networks (GNNs) and Manifold-valued Neural Networks (MVNs). Topology and Shape Analysis, which examines topological and geometric data properties, is another field that can be regarded as a subset of geometric deep learning.

Pineda et al. (2023) apply geometric deep learning to the analysis of dynamical process. For their mechanical interpretation and connection to biological functions, dynamical processes in living systems can be characterised in order to gain valuable information. It is now possible to capture the movements of cells, organelles, and individual molecules at several spatiotemporal scales in physiological settings to recent advancements in microscopy techniques. However, the capture of microscopic image sequences still lags behind the automated analysis of dynamics happening in crowded and complicated situations. The authors in the study mentioned in the reference (Pineda et al., 2023) offer a methodology built on geometric deep learning that successfully estimates dynamical properties with accuracy in a variety of biologically relevant settings. This deep learning strategy makes the use of an attention-based graph neural network (see in Table 1 for some literature reference on attention graphs). This network can do a variety of things, such as to convert coordinates into trajectories and infer local and global dynamic attributes, by processing object features with geometric *priors*.

Drug repositioning is another growing application domain of geometric deep learning on graphs. Currently, drug repositioning uses artificial intelligence tools to find new markers of authorised medications. The non-Euclidean nature of biological network data, however, is not well accounted for the majority of drug repositioning computational approaches. To solve this issue, Zhao et al. (2022) developed a deep learning system called DDAGDL. It uses geometric deep learning over heterogeneous information networks to predict drug-drug associations. By cleverly projecting drugs and diseases that include geometric prior knowledge of network topology in a non-Euclidean domain onto a latent feature space, DDAGDL can take advantage of complex biological information to learn the feature representations of pharmaceuticals and disorders. The authors in the study by Zhao et al. (2022) showed that according to experimental findings, DDAGDL may recognise high-quality candidates for breast neoplasms and Alzheimer's dementia that have already been described by published studies.

Another application of considerable interest in the medical and biological fields of geometric deep learning was proposed by Das et al. (2022). The authors start from the consideration that drug-virus interactions should be investigated in order to prepare for potential new forms of viruses and variants and rapidly generate medications or vaccinations against potential viruses. Despite expensive and time-consuming experimental procedures, geometric deep learning is a way that can be utilised to make this process faster and cheaper. Das et al. (2022) offered a new model based on geometric deep learning for predicting drug-virus interactions against COVID-19. First, in the SMILES molecular structure representation (Weininger, 1988), Das et al. employ antiviral medication data to generate features and better define the structure of chemical species. Then, the data are turned into a molecular representation, which is subsequently translated into a graphical structure that the geometrical deep learning model can understand.

Exploiting the idea that a molecule can be represented as a graph whose vertices correspond to the atoms of the compound and edges correspond to chemical bonds, geometric deep learning has been also applied for the prediction of molecular structure in structural chemistry and drug design (Hop et al., 2018; David et al., 2020; Atz et al., 2021). Geometric deep learning has been also applied to macromolecular structure (i.e., molecular graph) in structure-based drug design. Structure-based drug design identifies appropriate ligands by utilising the three-dimensional geometric information of macromolecules such as proteins or nucleic acids. An overview of recent geometric deep learning applications in bioorganic and medicinal chemistry, emphasising its promise for structure-based drug discovery and design, is presented by Isert et al. (2023). The focus of this overview is on predicting molecular properties, ligand binding site and posture prediction, and structure-based *de novo* molecular design. Similarly, geometric deep learning has been applied on molecular graph for molecular crystal structure prediction in mentioned in the study by Kilgour et al. (2023) and the RNA molecular structure prediction mentioned in the study by Townshend et al. (2021).

Although not directly relevant to the conception of the structure of a molecule as a graph, an interesting recent application of geometric deep learning to biomolecular data, which contributes to create knowledge about the interaction networks between proteins and other biomolecules, is the study by Gainza et al. (2019). The rationale of the authors in this study is that it is still difficult in biology to predict interactions between proteins and other biomolecules merely based on the structure. On the basis of their study, the experimental well-consolidated knowledge is that the molecular surface, a high-level illustration of protein structure, shows the patterns of chemical and geometric properties that uniquely identify a protein's modes of interaction with other biomolecules. Consequently, Gainza et al. hypothesized that independent of their evolutionary background, proteins involved in related interactions may have comparable signatures. Although fingerprints might be challenging to understand visually, they can be learned from big datasets. To identify fingerprints that are crucial for particular biomolecular interactions, the authors introduced MaSIF (molecular surface interaction fingerprinting), a conceptual framework based on a geometric deep learning algorithm. MaSIF can predict protein pockets with ligands, protein-protein interaction sites, and rapid scanning of protein surfaces for protein-protein complex prediction.

Townshend et al. (2021) pointed out that the intricate three-dimensional configurations that RNA molecules take on are challenging to measure experimentally or anticipate computationally. The analysis of the RNA structure is crucial to find medications to treat diseases that are currently incurable. A machine-learning technique proposed by Townshend et al. developed a geometric deep learning technique to enhance the prediction of RNA structures. Interestingly, the authors highlighted an important feature of geometric deep learning, namely, the flexibility of this approach to be suitably adapted to work with a smaller amount of training data than that required by traditional deep learning techniques.

Finally, we mention the use of geometric deep learning techniques in neuroimaging and the study of the brain connectome (Gurbuz and Rekik, 2020; Huang et al., 2021; Williams et al., 2021), as well as the study on (i) the relationship of human brain structure to cognitive function (Wu et al., 2022), (ii) the topographic heterogeneity of cortical organisation as a necessary step toward precision modelling of neuropsychiatric disorders (Williams et al., 2021), (iii) brain aging (Besson et al., 2022).

### 3.2 Latent geometry and graph comparison

To compare graphs, it is necessary to define a distance metric between graphs. This is a very complex undertaking that necessitates balancing interpretability, computational efficiency, and outcome effectiveness - all of which are frequently dependent on the particular application area. It should come as no surprise that there is a tonne of literature on this subject and that many various approaches have been suggested (Wills and Meyer, 2020). Of these three categories of methods for comparing graphs, the most closely related to latent geometry is the category of methods based on spectral analysis of the weighted adjacency matrix or Laplacian matrix, since the latent geometry of the network is derived from this. The rationale behind the use of spectral methods for graph comparison is that by comparing spectra provide metrics for comparing networks since the spectrum of a network's representation matrix (an adjacency or Laplacian matrix) contains information about its structure. In particular, the authors of NetSLD made explicit the relationship between weighted graph adjacency or Laplacian matrix and latent geometry through metaphors introduced by Günthard and Primas (1956) and Kac (1966). Spectral graph theory is effective in the comparison of 3D objects—said Tsitsulin et al. (2018)—but graphs lack a fixed form, yet 3D objects have an exact low-dimensional shape. However, a graph can be thought of as a geometric entity. Günthard and Primas (1956) posed the initial inquiry, “Can one hear the shape of a drum?” Kac (1966) elegantly posed the same query “To what extent may a graph (or, in general, a manifold) be determined by its spectrum?”. Since then, research has revealed that certain graphs are dictated by their spectrum and that isospectral graphs typically are isometric. Consequently, spectral graph theory provides a foundation for graph comparison.

Learning the latent geometry of a network also proves to be very useful in graph comparison because it can help address some of the challenges that this task presents. We summarise the main ones here and then explain how learning latent geometry can prove useful. First, graph comparison must not care about the order in which nodes are displayed; this is known as *permutation -invariance*. Second, a good method of graph comparison would make it possible to compare graphs both locally (representing, for example, the variations in atomic bonds between chemical compounds) and globally or communally (recording, for example, the various topologies of social networks). This ability is referred to as *scale-adaptivity*. Third, it would identify structural similarities regardless of network size (e.g., determining the similarity of two criminal networks of various sizes). This ability is referred to as *size-invariance*.

The research in graph comparison is very active today [see the recent review by Tantardini et al. (2019)], because—as Tsitsulin et al. (2018) highlights—there is not a method for graph comparison that meets all three of these criteria. In addition to these standards of quality, a workable method of graph comparison should be effectively computable. After preprocessing, it is suitable to do graph analytics jobs that frequently need pairwise graph comparisons inside a large collection of graphs in constant time. Unfortunately, current techniques perform considerably worse in this regard. Graph edit distance (GED) is a widely used graph distance; machine learning has utilised graph edit distance to compare objects when the objects are represented as attributed graphs rather than vectors. The GED is typically used in these situations to determine the distance between attributed graphs. GED is defined as the smallest number of edit operations (such as the deletion, insertion, and replacement of nodes and edges) required to change one graph into another (Serratosa, 2021). GED is NP-hard and APX-hard to compute (Lin, 1994); significant research in GED-based graph comparison has not been able to ignore this fact [see a concise but comprehensive state of the art on this in the study by Tsitsulin et al. (2018)]. Similar to GED, graph kernel approaches (Borgwardt et al., 2020; Kriege et al., 2020) do not explicitly represent the graph and and they do not scale well (Tsitsulin et al., 2018). Identifying the challenges in meeting the requirements of efficiency, scalability, size, and permutation invariance, Tsitsulin et al. moved the issue to the spectral realm and provides an evocative metaphor to clarify their process. Heating the nodes of the graph and tracking the heat spread over time is a metaphor. The idea of a system of masses for the graph's nodes and springs for its edges is another helpful metaphor. The authors (Tsitsulin et al., 2018) claimed that in both scenarios, the entire procedure embodies more global information with time and defines the graph in an efficient permutation-invariant way. They developed the NetLSD that summarises the features of the (undirected, unweighted) graph by a vector derived from the solution of the “heat equation” ∂*u*_*t*_/∂ = −*Lu*_*t*_, where *u*_*t*_ is an *n*-dimensional vector and = *I*−*D*^−1/2^*AD*^−1/2^ is the normalised Laplacian matrix. Since *L* is symmetric, it can be written as *L* = Φ*ΛΦ*^⊤^, where Λ is a diagonal matrix of sorted eigenvalues λ_1_ ≤ λ_2_ ≤ ⋯ ≤ λ_*n*_ of which ϕ_1_, ϕ_2_, …, ϕ_*n*_ are the corresponding eigenvectors. Hence, the closed-form solution is given by the *n*×*n* “heat kernel” matrix


$$\begin{array}{l} H_{t}=e^{-Lt}=\Phi e^{-\Lambda t}\Phi^{\text{T}} \end{array}$$

whose entry (_*H*_*t*_)*ij*_ is the heat transferred from node *i* to *j* at time *t*.

NetLSD summarises the graph representation in the heat trace signature as follows:


$$\begin{array}{l} h\left(G\right)={\left\{h_{t}\right\}}_{t>0}=\mathrm{trace}\left(H_{t}\right) \end{array}$$

Finally, the continuous-time function *h*_*t*_ is converted into a finite-dimensional vector by sampling over a suitable time interval, and the distance between two networks *G*_1_, *G*_2_ is taken as the *L*_2_-norm of the vector difference between *h*(*G*_1_) and *h*(*G*_2_). The time complexity of NetLSD is *O*(*n*^3^), if the full eigen-decomposition of the Laplacian is carried out. Specifically, the heat or wave kernel of the Laplacian spectrum is inherited by the compact signature that NetLSD derives, thus “it hears the shape of a graph”—said Tsitsulin et al. (2018).

At the conclusion of this section, some general comments on the spectral methods are made. Despite their use of ease and rigorous theoretical foundation, these methods exhibit some limitations, such as cospectrality between graphs, reliance on matrix representation, and abnormal sensitivity, wherein slight alterations in the graph's structure can result in significant changes in the spectrum (Tantardini et al., 2019) and *vice versa*. However, it is reasonable to think that strategies to manage the sensitivity to noise or perturbations will not be long in coming, given the numerous advantages that spectral methods offer compared with their limitations, first of all the possibility that these methods offer to efficiently satisfy the three main desiderate of graph comparison as NetLSD has demonstrated.

## 4 Challenges and future developments

A comprehensive review by Cai et al. (2018) outlines the challenges of graph embedding. In that review, the authors emphasized how the challenges of embedding a graph rely on the issue formulation, which entails embedding input and embedding output. The input to graph embedding algorithms according to Cai et al. can be categorized into four groups: homogeneous, heterogeneous, with auxiliary information, and graph built from non-relational data. The task of graph embedding is complicated by the fact that different forms of embedding input require different information to be kept in the embedded space. For instance, when embedding a graph with only structure information, the goal is to preserve the connections between the nodes. The auxiliary information, however, gives graph properties from various perspectives and may therefore also be taken into account during the embedding for a graph that has node label or attribute information. In contrast to the given and fixed embedding input, the embedding output is task driven – said Cai et al. (2018). For instance, node embedding depicts nearby nodes as comparable vectors. Node-related activities such as node classification, node clustering can benefit from node embedding. However, in some circumstances, the tasks may pertain to a finer level of a network, such as node pairs, subgraphs, or the entire graph. Finding an appropriate embedding output type for the application of interest is thus the first hurdle in terms of embedding output. Node embedding, edge embedding, hybrid embedding, and whole-graph embedding are the four output categories commonly used to classify graph embedding results. Cai et al. (2018) then continue by asserting that distinct output granularities are faced with various difficulties and have distinct requirements for an optimal embedding. A successful node embedding, for instance, maintains the resemblance to its surrounding nodes in the embedded space. A decent whole-graph embedding, on the other hand, depicts a full graph as a vector in order to maintain the graph's level of similarity.

Since the output of embedding is task-driven, we can rightly imagine that the quality of the output is also contextual to the task. Consequently, any procedure for evaluating and validating graph embedding must necessarily refer to particular tasks and contexts. Regarding applications in biology of graph embedding, we mention here a study by Yue et al. (2019) that compared the performance of embedding methods with the purpose of link prediction. Three significant biomedical link prediction tasks–drug-disease association (DDA), drug-drug interaction (DDI), and protein-protein interaction (PPI) prediction–as well as two node classification tasks–classification of medical terms according to their semantic types and protein function prediction–are the subjects of the systematic comparison of 11 representative graph embedding methods as shown in the study mentioned in the reference (Yue et al., 2019). According to Yue et al., the results of the test show that current graph embedding techniques are effective and deserve additional consideration in biological graph analysis in future. Recent graph embedding methods achieve competitive performance without using any biological features, and the learned embeddings can be treated as complementary representations for the biological features when compared with three state-of-the-art methods, namely, DDAs, DDIs, and protein function predictions. The comparative review by Yue et al. finally offers broad recommendations for choosing graph embedding algorithms and configuring their hyper-parameters for various biomedical objectives.

Graph embedding is proved to be of particular importance for the prediction of links in a network and, more generally, in all declinations of network inference (see Saint-Antoine and Singh, 2020 for a review on network inference challenges), a pivotal task in systems biology and, more specifically, gene regulatory network inference and single cell biology. In this regard, Hetzel et al. (2021) present a viewpoint on learning graph representations that is specifically inspired by the uses and difficulties of (i) single-cell biology; (ii) network inference, and (iii) current developments in spatial molecular profiling. Hetzel et al. (2021) state that single-cell RNA sequencing provides previously unattainable levels of resolution and scale for measuring gene expression and enables the investigation of cellular characteristics. Graphs provide a natural representation of the system in this setting, both as gene- and cell-centric. Thus, we foresee the possibility of obtaining highly informative results from graph embedding of such accurate and good quality data.

Finally, an additional challenge that graph embedding methods and geometric deep learning face are the management of noise in the data. The quality of the input data, as determined by the value of the experimental uncertainties on edge weights and/or structural noise that plague them, and the sparsity of the data are two parameters that affect the quality and thus the reliability of embedding results and consequently their geometric deep learning. Some studies are already tried to address these challenges (Zhang et al., 2012; Pujara et al., 2017; Fox and Rajamanickam, 2019; Okuno and Shimodaira, 2019; Cheng et al., 2020; Hong et al., 2021; Xia et al., 2023).

## 5 Conclusion

In this mini-review, we have reviewed and collected an abundance of literature on graph embedding techniques and geometric deep learning methods of great relevance for graph mining of biological networks and/or biophysical systems that can be represented as networks/graphs. Since non-Euclidean data cannot be effectively represented using conventional deep learning techniques, geometric deep learning offers a significant advancement in the field of machine learning. Deep learning models in applied artificial intelligence perform appropriately with Euclidean data, but they performed with non-Euclidean data. Because it is usual to find data of this type in biology, biochemistry, and biophysics, this is a significant problem which is the rationale of the research studies in geometric deep learning. Graph embedding and geometric deep learning are not two separate methods but can operate in sequence since graph embedding can provide geometric information about nodes and/or arcs, which can then be used by geometric deep learning for more complete, reliable, and accurate analysis and inference of new knowledge.

## Author contributions

PL: Conceptualization, Funding acquisition, Investigation, Writing—original draft, Writing—review & editing. ML: Investigation, Writing—original draft, Writing—review & editing.

## References

[B1] Abbas K. Abbasi A. Dong S. Niu L. Chen L. Chen B. (2023). A novel temporal network-embedding algorithm for link prediction in dynamic networks. Entropy 25, 257. 10.3390/e25020257 36832623 PMC9955760

[B2] Alanis-Lobato G. Mier P. Andrade-Navarro M. (2018). The latent geometry of the human protein interaction network. Bioinformatics 34, 2826–2834. 10.1093/bioinformatics/bty206 29635317 PMC6084611

[B3] Amara A. Hadj Taieb M. A. Ben Aouicha M. (2021). Network representation learning systematic review: Ancestors and current development state. Mach. Learn. Appl. 6, 100130. 10.1016/j.mlwa.2021.100130

[B4] Archdeacon D. (1990). “The complexity of the graph embedding problem,” in Topics in Combinatorics and Graph Theory, eds R. Bodendiek and R. Henn (Heidelberg: Physica-Verlag). 10.1007/978-3-642-46908-4_6

[B5] Atz K. Grisoni F. Schneider G. (2021). Geometric deep learning on molecular representations. Nat. Mach. Intellig. 3, 1023–1032. 10.1038/s42256-021-00418-8

[B6] Bagrow J. P. Bollt E. M. (2019). An information-theoretic, all-scales approach to comparing networks. Appl. Netw. Sci. 4, 1. 10.1007/s41109-019-0156-x

[B7] Baldi P. (2012). “Autoencoders, unsupervised learning, and deep architectures,” in Proceedings of ICML Workshop on Unsupervised and Transfer Learning, Vol. 27, eds I. Guyon, G. Dror, V. Lemaire, G. Taylor, and D. Silver (Bellevue, WA: PMLR), 37–49. Available online at: https://proceedings.mlr.press/v27/baldi12a.html

[B8] Béres F. Kelen D. M. Pálovics R. Benczúr A. A. (2019). Node embeddings in dynamic graphs. Appl. Netw. Sci. 4, 1. 10.1007/s41109-019-0169-5

[B9] Besson P. Rogalski E. Gill N. P. Zhang H. Martersteck A. Bandt S. K. (2022). Geometric deep learning reveals a structuro-temporal understanding of healthy and pathologic brain aging. Front. Aging Neurosci. 14, 895535. 10.3389/fnagi.2022.895535 36081894 PMC9445244

[B10] Bianconi G. Rahmede C. (2017). Emergent hyperbolic network geometry. Sci. Rep. 7, 974. 10.1038/srep41974 28167818 PMC5294422

[B11] Boguñá M. Bonamassa I. Domenico M. D. Havlin S. Krioukov D. Serrano M.. (2021). Network geometry. Nat. Rev. Phys. 3, 114–135. 10.1038/s42254-020-00264-4

[B12] Bombelli L. Lee J. Meyer D. Sorkin R. D. (1987). Space-time as a causal set. Phys. Rev. Lett. 59, 521–524. 10.1103/PhysRevLett.59.521 10035795

[B13] Borgwardt K. Ghisu E. Llinares-López F. O'Bray L. Rieck B. (2020). Graph kernels: State-of-the-art and future challenges. Trends Mach. Learn. 13 531–712. 10.1561/2200000076

[B14] Bronstein M. M. Bruna J. Cohen T. Veličković P. (2021). Geometric deep learning: grids, groups, graphs, geodesics, and gauges. ArXiv. abs/2104.13478. Available online at: https://api.semanticscholar.org/CorpusID:233423603

[B15] Bronstein M. M. Bruna J. LeCun Y. Szlam A. Vandergheynst P. (2017). Geometric deep learning: going beyond euclidean data. IEEE Signal Process. Mag. 34, 18–42. 10.1109/MSP.2017.2693418

[B16] Cai H. Zheng V. W. Chang K. C.-C. (2018). A comprehensive survey of graph embedding: problems, techniques, and applications. IEEE Trans. Knowl. Data Eng. 30, 1616–1637. 10.1109/TKDE.2018.2807452

[B17] Cao J. Lin X. Guo S. Liu L. Liu T. Wang B. (2021). “Bipartite graph embedding via mutual information maximization,” in Proceedings of the 14th ACM International Conference on Web Search and Data Mining (New York, NY: Association for Computing Machinery), 635–643. 10.1145/3437963.3441783

[B18] Cao S. Lu W. Xu Q. (2015). “Grarep: Learning graph representations with global structural information,” in Proceedings of the 24th ACM International on Conference on Information and Knowledge Management, CIKM '15 (New York, NY: Association for Computing Machinery), 891–900.

[B19] Cao S. Lu W. Xu Q. (2016a). “Deep neural networks for learning graph representations,” in Proceedings of the Thirtieth AAAI Conference on Artificial Intelligence (Phoenix, AZ: AAAI Press), 1145–1152.

[B20] Cao W. Yan Z. He Z. He Z. (2020). A comprehensive survey on geometric deep learning. IEEE Access 8, 35929–35949. 10.1109/ACCESS.2020.2975067

[B21] Cao X. Zheng Y. Shi C. Li J. Wu B. (2016b). “Link prediction in schema-rich heterogeneous information network,” in Pacific-Asia Conference on Knowledge Discovery and Data Mining.

[B22] Chakraborty R. Bouza J. Manton J. H. Vemuri B. C. (2022). ManifoldNet: A deep neural network for manifold-valued data with applications. IEEE Trans. Pattern Anal. Mach. Intell. 44, 799–810. 10.1109/TPAMI.2020.3003846 32750791

[B23] Chang J. Lan Z. Cheng C. Wei Y. (2020). “Data uncertainty learning in face recognition,” in 2020 IEEE/CVF Conference on Computer Vision and Pattern Recognition (CVPR), Seattle, WA (Manhattan, NY: IEEE), 5709–5718. 10.1109/CVPR42600.2020.00575

[B24] Charles R. Q. Su H. Kaichun M. Guibas L. J. (2017). “PointNet: Deep learning on point sets for 3d classification and segmentation,” in 2017 IEEE Conference on Computer Vision and Pattern Recognition (CVPR). Manhattan, NY: IEEE.

[B25] Chen H. Perozzi B. Hu Y. Skiena S. (2018). “Harp: Hierarchical representation learning for networks,” in Proceedings of the Thirty-Second AAAI Conference on Artificial Intelligence and Thirtieth Innovative Applications of Artificial Intelligence Conference and Eighth AAAI Symposium on Educational Advances in Artificial Intelligence, AAAI'18/IAAI'18/EAAI'18. Washington D.C.: AAAI Press.

[B26] Chen J. Fang C. Zhang X. (2022). Global attention-based graph neural networks for node classification. Neur. Proc. Lett. 55, 4127–4150. 10.1007/s11063-022-11032-z

[B27] Chen J. Kanchi S. P. Kanevsky A. (1993). “On the complexity of graph embeddings,” in Lecture Notes in Computer Science. Cham: Springer Berlin Heidelberg, 234–245.

[B28] Chen M. Yang Q. Tang X. (2007). “Directed graph embedding,” in Proceedings of the 20th International Joint Conference on Artifical Intelligence, IJCAI'07. San Francisco, CA, USA: Morgan Kaufmann Publishers Inc, 2707–2712.

[B29] Cheng K. Zhu Y. Zhang M. Sun Y. (2020). Noi gan *NOISE aware knowledge graph embedding with gan*?°

[B30] Chung F. (2006). The diameter and laplacian eigenvalues of directed graphs. Electr. J. Combinator. 13, 1. 10.37236/1142

[B31] Clough J. R. Evans T. S. (2017). Embedding graphs in lorentzian spacetime. PLoS ONE 12, e0187301. 10.1371/journal.pone.0187301 29107967 PMC5673185

[B32] Das B. Kutsal M. Das R. (2022). A geometric deep learning model for display and prediction of potential drug-virus interactions against SARS-CoV-2. Chemometrics and Intellig. Lab. Syst. 229, 104640. 10.1016/j.chemolab.2022.104640 36042844 PMC9400382

[B33] David L. Thakkar A. Mercado R. Engkvist O. (2020). Molecular representations in AI-driven drug discovery: a review and practical guide. J. Cheminform. 12, 5. 10.1186/s,13321-020-00460-5 33431035 PMC7495975

[B34] DGL LifeSci (2020). Readout for Computing Graph Representations; DGL-LifeSci 0.3.1 documentation – *lifesci.dgl.ai*. Available online at: https://lifesci.dgl.ai/api/model.readout.html#::text=After%20updating%20node%2Fedge%20representations,of%20updated%20node%2Fedge%20representations (accessed July 9, 2023).

[B35] East R. (2023). Introduction to Geometric Quantum Machine Learning | *PennyLane Demos*. Available online at: https://pennylane.ai/qml/demos/tutorial_geometric_qml (accessed September 18, 2023).

[B36] Etaiwi W. Awajan A. (2023). Semanticgraph2vec: Semantic graph embedding for text representation. Array 17, 100276. 10.1016/j.array.2023.100276

[B37] Faisal F. E. Newaz K. Chaney J. L. Li J. Emrich S. J. Clark P. L.. (2017). GRAFENE: Graphlet-based alignment-free network approach integrates 3d structural and sequence (residue order) data to improve protein structural comparison. Sci. Rep. 7, 1. 10.1038/s41598-017-14411-y 29097661 PMC5668259

[B38] Fang G. Zeng F. Li X. Yao L. (2021). Word2vec based deep learning network for DNA n4-methylcytosine sites identification. Procedia Comput. Sci. 187, 270–277. 10.1016/j.procs.2021.04.062

[B39] Fox J. Rajamanickam S. (2019). How Robust are Graph Neural Networks to Structural Noise?

[B40] Francis D. P. Raimond K. (2020). Major advancements in kernel function approximation. Artif. Intellig. Rev. 54, 843–876. 10.1007/s10462-020-09880-z

[B41] Gainza P. Sverrisson F. Monti F. Rodolà E. Boscaini D. Bronstein M. M.. (2019). Deciphering interaction fingerprints from protein molecular surfaces using geometric deep learning. Nat. Methods 17, 184–192. 10.1038/s41592-019-0666-6 31819266

[B42] Gao K. Zhang J. Zhou C. (2019). Semi-supervised graph embedding for multi-label graph node classification. *ArXiv*. abs/1907.05743. Available online at: https://api.semanticscholar.org/CorpusID:196470841

[B43] Gauvin L. Panisson A. Cattuto C. (2014). Detecting the community structure and activity patterns of temporal networks: A non-negative tensor factorization approach. PLoS ONE 9, e86028. 10.1371/journal.pone.0086028 24497935 PMC3908891

[B44] Gharavi E. Gu A. Zheng G. Smith J. P. Cho H. J. Zhang A.. (2021). Embeddings of genomic region sets capture rich biological associations in lower dimensions. Bioinformatics 37, 4299–4306. 10.1093/bioinformatics/btab439 34156475 PMC8652032

[B45] Giamphy E. Guillaume J.-L. Doucet A. Sanchis K. (2023). A survey on bipartite graphs embedding. Soc. Netw. Analy. Mini. 13, 1. 10.1007/s13278-023-01058-z

[B46] Goyal P. Ferrara E. (2018). Graph embedding techniques, applications, and performance: a survey. Knowl.-Based Syst. 151, 78–94. 10.1016/j.knosys.2018.03.022

[B47] Grover A. Leskovec J. (2016). “Node2vec: Scalable feature learning for networks,” in Proceedings of the 22nd ACM SIGKDD International Conference on Knowledge Discovery and Data Mining, KDD '16. New York, NY, USA: Association for Computing Machinery, 855–864. 10.1145/2939672.2939754 PMC5108654 27853626

[B48] Günthard H. H. Primas H. (1956). Zusammenhang von Graphentheorie und MO-Theorie von Molekeln mit Systemen konjugierter Bindungen. Helv. Chim. Acta 39, 1645–1653. 10.1002/hlca.19560390623

[B49] Gurbuz M. B. Rekik I. (2020). “Deep graph normalizer: A geometric deep learning approach for estimating connectional brain templates,” in Medical Image Computing and Computer Assisted Intervention –MICCAI 2020. Cham: Springer International Publishing, 155–165.

[B50] Hasibi R. Michoel T. (2021). A graph feature auto-encoder for the prediction of unobserved node features on biological networks. BMC Bioinformat. 22, 3. 10.1186/s12859-021-04447-3 34706640 PMC8554915

[B51] Hensel F. Moor M. Rieck B. (2021). A survey of topological machine learning methods. Front. Artif. Intellig. 4, 155–165. 10.3389/frai.2021.155-165 34124648 PMC8187791

[B52] Hetzel L. Fischer D. S. Günnemann S. Theis F. J. (2021). Graph representation learning for single-cell biology. Curr. Opini. Syst. Biol. 28, 100347. 10.1016/j.coisb.2021.05.008

[B53] Holme P. Saramäki J. (2012). Temporal networks. Phys. Rep. 519, 97–125. 10.1016/j.physrep.2012.03.001

[B54] Hong H. Guo H. Lin Y. Yang X. Li Z. Ye J. (2020). An attention-based graph neural network for heterogeneous structural learning. Proc. Innov. Appl. Artif. Intell. Conf. 34, 4132–4139. 10.1609/aaai.v34i04.5833

[B55] Hong Y. Bu C. Wu X. (2021). High-quality noise detection for knowledge graph embedding with rule-based triple confidence,” in PRICAI *2021: Trends in Artificial Intelligence*. Cham: Springer International Publishing, 572–585.

[B56] Hop P. Allgood B. Yu J. (2018). Geometric deep learning autonomously learns chemical features that outperform those engineered by domain experts. Mol. Pharm. 15, 4371–4377. 10.1021/acs.molpharmaceut.7b01144 29863875

[B57] Hostallero D. E. Li Y. Emad A. (2022). Looking at the BiG picture: incorporating bipartite graphs in drug response prediction. Bioinformatics 38, 3609–3620. 10.1093/bioinformatics/btac383 35674359

[B58] Huang X. Li J. Hu X. (2017). “Label informed attributed network embedding,” in Proceedings of the Tenth ACM International Conference on Web Search and Data Mining, WSDM '17. New York, NY, USA: Association for Computing Machinery, 731–739.

[B59] Huang Z. Cai H. Dan T. Lin Y. Laurienti P. Wu G. (2021). Detecting brain state changes by geometric deep learning of functional dynamics on riemannian manifold. In Medical Image Computing and Computer Assisted Intervention –MICCAI 2021, pages 543-552. Springer International Publishing. 10.1007/978-3-030-87234-2_51

[B60] Isert C. Atz K. Schneider G. (2023). Structure-based drug design with geometric deep learning. Curr. Opin. Struct. Biol. 79, 102548. 10.1016/j.sbi.2023.102548 36842415

[B61] Jiao P. Guo X. Jing X. He D. Wu H. Pan S.. (2022). Temporal network embedding for link prediction via VAE joint attention mechanism. IEEE Trans. Neural Netw. Learn. Syst. 33, 7400–7413. 10.1109/TNNLS.2021.3084957 34106869

[B62] Kac M. (1966). Can one hear the shape of a drum? Am. Mathematical Monthly 73, 1–23. 10.1080/00029890.1966.1197091517749416

[B63] Kamiński B. Pra P. Théberge F. (2019). An unsupervised framework for comparing graph embeddings. J. Complex Netw. 8, 5. 10.1093/comnet/cnz043

[B64] Karpukhin I. Dereka S. Kolesnikov S. (2022). GitHub - tinkoff-ai/probabilistic-embeddings: “Probabilistic Embeddings Revisited” Paper Official Repository — github.com. Available online at: https://github.com/tinkoff-ai/probabilistic-embeddings (accessed October 20, 2023).

[B65] Kehr B. Trappe K. Holtgrewe M. Reinert K. (2014). Genome alignment with graph data structures: a comparison. BMC Bioinformat. 15, 99. 10.1186/1471-2105-15-99 24712884 PMC4020321

[B66] Khosla M. Leonhardt J. Nejdl W. Anand A. (2020). “Node representation learning for directed graphs,” in Machine Learning and Knowledge Discovery in Databases - European Conference, ECML PKDD 2019, Proceedings, Vol. 11906 (Lecture Notes in Computer Science), eds U. Brefeld, E. Fromont, A. Hotho, A. Knobbe, M. Maathuis, and C. Robardet (Springer International Publishing), 395–411. 10.1007/978-3-030-46150-8_24

[B67] Kilgour M. Rogal J. Tuckerman M. (2023). Geometric deep learning for molecular crystal structure prediction. J. Chem. Theory Comput. 19, 4743–4756. 10.1021/acs.jctc.3c00031 37053511 PMC10373482

[B68] Kim M. Baek S. H. Song M. (2018). Relation extraction for biological pathway construction using node2vec. BMC Bioinformat. 19, 8. 10.1186/s12859-018-2200-8 29897325 PMC5998757

[B69] Kleinberg J. M. (1999). Authoritative sources in a hyperlinked environment. J. ACM. 46, 604–632. 10.1145/324133.324140

[B70] Kolář M. (2013). “Graph alignment, protein interaction networks,” in Encyclopedia of Systems Biology. New York: Springer New York, 861–865.

[B71] Koutra D. Shah N. Vogelstein J. T. Gallagher B. Faloutsos C. (2016). Deltacon: Principled massive-graph similarity function with attribution. ACM Trans. Knowl. Discov. Data 10, 3. 10.1145/2824443

[B72] Kriege N. M. Johansson F. D. Morris C. (2020). A survey on graph kernels. Appl. Netw. Sci. 5, 3. 10.1007/s41109-019-0195-3

[B73] Krioukov D. (2016). Clustering implies geometry in networks. Phys. Rev. Lett. 116, 2. 10.1103/PhysRevLett.116.208302 27258887

[B74] Krioukov D. Papadopoulos F. Kitsak M. Vahdat A. Bogu ná M. (2010). Hyperbolic geometry of complex networks. Physical Review E 82, 3. 10.1103/PhysRevE.82.036106 21230138

[B75] Law M. (2021). “Ultrahyperbolic neural networks,” in Advances in Neural Information Processing Systems, eds. M. Ranzato, A. Beygelzimer, Y. Dauphin, P. Liang, and Y. W. Vaughan. New York: Curran Associates, Inc, 22058–22069.

[B76] Law M. T. Lucas J. (2023). “Spacetime representation learning,” in Proceedings of The Eleventh Inter International Conference on Learning Representations, ICLR (Kigali). Available online at: OpenReview.net

[B77] Law M. T. Stam J. (2020). “Ultrahyperbolic representation learning,” in Proceedings of the 34th International Conference on Neural Information Processing Systems, NIPS'20. Red Hook, NY, USA: Curran Associates Inc.

[B78] Lecca P. Re A. (2022). “Checking for non-euclidean latent geometry of biological networks,” in 2022 IEEE International Conference on Bioinformatics and Biomedicine (BIBM). Las Vegas, NV: IEEE.

[B79] Lempel R. Moran S. (2001). SALSA. ACM Trans. Informat. Syst. 19, 131–160. 10.1145/382979.383041

[B80] Li H. Li L. Xv G. Lin C. Li K. Jiang B. (2021a). SPEX: A generic framework for enhancing neural social recommendation. ACM Trans. Informat. Syst. 40, 1–33. 10.1145/3473338

[B81] Li J. Zhu J. Zhang B. (2016a). “Discriminative deep random walk for network classification,” in Proceedings of the 54th Annual Meeting of the Association for Computational Linguistics (Volume 1: Long Papers). Berlin, Germany: Association for Computational Linguistics, 1004–1013.

[B82] Li J. Zhu J. Zhang B. (2016b). “Discriminative deep random walk for network classification,” in Proceedings of the 54th Annual Meeting of the Association for Computational Linguistics (Volume 1: Long Papers). Berlin, Germany: Association for Computational Linguistics, 1004–1013.

[B83] Li X. Liu Y. Wang Y. (2021b). “Object detection in omnidirectional images based on spherical cnn,” in 2021 7th IEEE International Conference on Network Intelligence and Digital Content (IC-NIDC). Beijing: IEEE, 269–273.

[B84] Liao L. He X. Zhang H. Chua T.-S. (2018). Attributed social network embedding. IEEE Trans. Knowl. Data Eng. 30, 2257–2270. 10.1109/TKDE.2018.2819980

[B85] Liao S. Liang S. Meng Z. Zhang Q. (2021). “Learning dynamic embeddings for temporal knowledge graphs,” in Proceedings of the 14th ACM International Conference on Web Search and Data Mining. New York: ACM.

[B86] Lin C.-L. (1994). “Hardness of approximating graph transformation problem,” in Algorithms and Computation. Berlin: Springer Berlin Heidelberg, 74–82.

[B87] Liu D. Ru Y. Li Q. Wang S. Niu J. (2020). Semisupervised community preserving network embedding with pairwise constraints. Complexity 2020, 1–14. 10.1155/2020/7953758

[B88] Liu M. Quan Z.-W. Wu J.-M. Liu Y. Han M. (2022). Embedding temporal networks inductively via mining neighborhood and community influences. Appl. Intellig. 52, 16069–16088. 10.1007/s10489-021-03102-x

[B89] Liu Q. Dong Z. Wang E. (2018). Cut based method for comparing complex networks. Sci. Rep. 8, 5. 10.1038/s41598-018-21532-5 29572479 PMC5865141

[B90] Ma Y. Zhang H. Jin C. Kang C. (2023). Predicting lncRNA-protein interactions with bipartite graph embedding and deep graph neural networks. Front. Genet. 14. 10.3389/fgene.2023.1136672 36845380 PMC9948011

[B91] Magai G. Ayzenberg A. (2022). Topology and geometry of data manifold in deep learning. arXiv preprint arXiv:2204.08624.

[B92] Makarov I. Savchenko A. Korovko A. Sherstyuk L. Severin N. Kiselev D.. (2022). Temporal network embedding framework with causal anonymous walks representations. PeerJ. Comp. Sci. 8, e858. 10.7717/peerj-cs.858 35174275 PMC8802774

[B93] Manoochehri H. E. Nourani M. (2020). Drug-target interaction prediction using semi-bipartite graph model and deep learning. BMC Bioinformat. 21, 6. 10.1186/s12859-020-3518-6 32631230 PMC7336396

[B94] Meilă M. Pentney W. (2007). “Clustering by weighted cuts in directed graphs,” in Proceedings of the 2007 SIAM International Conference on Data Mining. Philadelphia, PA: Society for Industrial and Applied Mathematics.

[B95] Mena J. Pujol O. Vitri? J. (2020). Uncertainty-based rejection wrappers for black-box classifiers. IEEE Access 8, 101721–101746. 10.1109/ACCESS.2020.2996495

[B96] Misra A. (2019). Capsule Networks: The New Deep Learning Network. Available online at: https://towardsdatascience.com/capsule-networks-the-new-deep-learning-network-bd917e6818e8 (accessed July 8, 2023).

[B97] Mohan A. Pramod K. V. (2019). Network representation learning: models, methods and applications. SN Appl. Sci. 1, 9. 10.1007/s42452-019-1044-9

[B98] Mohan A. Pramod K. V. (2021). Temporal network embedding using graph attention network. Complex Intellig. Syst. 8, 13–27. 10.1007/s40747-021-00332-x

[B99] Nelson W. Zitnik M. Wang B. Leskovec J. Goldenberg A. Sharan R. (2019). To embed or not: network embedding as a paradigm in computational biology. Front. Genet. 10, 381. 10.3389/fgene.2019.00381 31118945 PMC6504708

[B100] Nguyen G. H. Lee J. B. Rossi R. A. Ahmed N. K. Koh E. Kim S. (2018). “Continuous-time dynamic network embeddings,” in Companion of the The Web Conference 2018 on The Web Conference 2018 - *WWW '18*. New York City: ACM Press.

[B101] Okuno A. Shimodaira H. (2019). “Robust graph embedding with noisy link weights,” in Proceedings of the Twenty-Second International Conference on Artificial Intelligence and Statistics, Vol. 89, eds. K. Chaudhuri, and M. Sugiyama (PMLR), 664–673. Available online at: https://proceedings.mlr.press/v89/okuno19b.html

[B102] Ömer Nebil Yaveroğlu Milenković T. Pržulj N. (2015). Proper evaluation of alignment-free network comparison methods. Bioinformatics 31, 2697–2704. 10.1093/bioinformatics/btv170 25810431 PMC4528624

[B103] Ou M. Cui P. Pei J. Zhang Z. Zhu W. (2016). “Asymmetric transitivity preserving graph embedding,” in Proceedings of the 22nd ACM SIGKDD International Conference on Knowledge Discovery and Data Mining, KDD '16. New York, NY, USA: Association for Computing Machinery, 1105–1114.

[B104] Öztürk H. Özgür A. Schwaller P. Laino T. Ozkirimli E. (2020). Exploring chemical space using natural language processing methodologies for drug discovery. Drug Discov. Today 25:689–705. 10.1016/j.drudis.2020.01.020 32027969

[B105] Pan S. Hu R. Fung S.-F. Long G. Jiang J. Zhang C. (2020). Learning graph embedding with adversarial training methods. IEEE Trans. Cybern. 50, 2475–2487. 10.1109/TCYB.2019.2932096 31484146

[B106] Pan S. Wu J. Zhu X. Zhang C. Wang Y. (2016). “Tri-party deep network representation,” in Proceedings of the Twenty-Fifth International Joint Conference on Artificial Intelligence, IJCAI'16. New York: AAAI Press, 1895–1901.

[B107] Pandhre S. Mittal H. Gupta M. Balasubramanian V. N. (2018). “STwalk,” in Proceedings of the ACM India Joint International Conference on Data Science and Management of Data. New York: ACM.

[B108] Papadopoulos F. Flores M. A. R. (2019). Latent geometry and dynamics of proximity networks. Physical Rev. E 100, 5. 10.1103/PhysRevE.100.052313 31870016

[B109] Pavlopoulos G. A. Kontou P. I. Pavlopoulou A. Bouyioukos C. Markou E. Bagos P. G. (2018). Bipartite graphs in systems biology and medicine: a survey of methods and applications. Gigascience 7, 4. 10.1093/gigascience/giy014 PMC6333914 29648623

[B110] Perozzi B. Al-Rfou R. Skiena S. (2014). “DeepWalk,” in Proceedings of the 20th ACM SIGKDD International Conference on Knowledge Discovery and Data Mining. New York, NY: Association for Computing Machinery.

[B111] Perozzi B. Kulkarni V. Skiena S. (2016). Walklets: Multiscale graph embeddings for interpretable network classification. ArXiv. abs/1605.02115.

[B112] Perrault-joncas D. Meila M. (2011). “Directed graph embedding: an algorithm based on continuous limits of laplacian-type operators,” in Advances in Neural Information Processing Systems, J. Shawe-Taylor, R. Zemel, P. Bartlett, F. Pereira, and K. Weinberger. New York: Curran Associates, Inc.

[B113] Pineda J. Midtvedt B. Bachimanchi H. Noé S. Midtvedt D. Volpe G.. (2023). Geometric deep learning reveals the spatiotemporal features of microscopic motion. Nat. Mach. Intellig. 5, 71–82. 10.1038/s42256-022-00595-0

[B114] Pujara J. Augustine E. Getoor L. (2017). “Sparsity and noise: Where knowledge graph embeddings fall short,” in Proceedings of the 2017 Conference on Empirical Methods in Natural Language Processing. New York: Association for Computational Linguistics.

[B115] Qiu J. Dong Y. Ma H. Li J. Wang K. Tang J. (2018). “Network embedding as matrix factorization: Unifying deepwalk, line, pte, and node2vec,” in Proceedings of the Eleventh ACM International Conference on Web Search and Data Mining, WSDM '18. New York, NY, USA: Association for Computing Machinery, 459–467.

[B116] Saint-Antoine M. M. Singh A. (2020). Network inference in systems biology: recent developments, challenges, and applications. Curr. Opin. Biotechnol. 63, 89–98. 10.1016/j.copbio.2019.12.002 31927423 PMC7308210

[B117] Salim A. Sumitra S. (2022). Spectral graph convolutional neural networks in the context of regularization theory. IEEE Trans. Neural Netw. Learn. Syst. 2022, 1–12. 10.1109/TNNLS.2022.3177742 35696484

[B118] Sato K. Oka M. Barrat A. Cattuto C. (2021). Predicting partially observed processes on temporal networks by Dynamics-Aware Node Embeddings (DyANE). EPJ Data Sci. 10, 22. 10.1140/epjds/s13688-021-00277-8

[B119] Saxena T. Xu D. (2021). Graph Alignment-Based Protein Comparison. Available online at: https://math.mit.edu/research/highschool/primes/materials/2020/Saxena-Xu.pdf (accessed October 17, 2023).

[B120] Scott O. B. Gu J. Chan A. E. (2022). Classification of protein-binding sites using a spherical convolutional neural network. J. Chem. Inf. Model. 62, 5383–5396. 10.1021/acs.jcim.2c00832 36341715 PMC9709917

[B121] Serratosa F. (2021). Redefining the graph edit distance. SN Comp. Sci. 2, 6. 10.1007/s42979-021-00792-5

[B122] Shen X. Chung F.-L. (2020). Deep network embedding for graph representation learning in signed networks. IEEE Trans. Cybern. 50, 1556–1568. 10.1109/TCYB.2018.2871503 30307885

[B123] Shi M. Tang Y. Zhu X. (2020). Mlne: Multi-label network embedding. IEEE Trans. Neural Netw. Learn. Syst 31, 3682–3695. 10.1109/TNNLS.2019.2945869 31722493

[B124] Sim A. Wiatrak M. Brayne A. Creed P. Paliwal S. (2021). “Directed graph embeddings in pseudo-riemannian manifolds,” in International Conference on Machine Learning. Honolulu: Hawaii Convention Center.

[B125] Singer U. Guy I. Radinsky K. (2019). “Node embedding over temporal graphs,” in Proceedings of the Twenty-Eighth International Joint Conference on Artificial Intelligence. Cape Town: International Joint Conferences on Artificial Intelligence Organization.

[B126] Sivakumar D. (2023). Introduction to Geometric Deep Learning - *Scaler Topic*. Available online at: https://www.scaler.com/topics/geometric-deep-learning/ (accessed July 6, 2023).

[B127] Soltanshahi M. A. Teimourpour B. Khatibi T. Zare H. (2022). Grar: a novel framework for graph alignment based on relativity concept. Expert Syst. Appl. 187, 115908. 10.1016/j.eswa.2021.115908

[B128] Song G. Zhang Y. Xu L. Lu H. (2022). Domain adaptive network embedding. IEEE Trans. Big Data 8, 1220–1232. 10.1109/TBDATA.2020.3034201

[B129] Su Y.-C. Grauman K. (2021). Learning spherical convolution for 360 recognition. IEEE Trans. Pattern Analy. Mach. Intellig. 44, 8371–8386. 10.1109/TPAMI.2021.3113612 34543192

[B130] Tang J. Qu M. Mei Q. (2015a). “PTE,” in Proceedings of the 21th ACM SIGKDD International Conference on Knowledge Discovery and Data Mining. New York: ACM.

[B131] Tang J. Qu M. Wang M. Zhang M. Yan J. Mei Q. (2015b). “Line: Large-scale information network embedding,” in Proceedings of the 24th International Conference on World Wide Web. Geneva: International World Wide Web Conferences Steering Committee.

[B132] Tang J. Qu M. Wang M. Zhang M. Yan J. Mei Q. (2015c). Line: Large-scale information network embedding,” in *Proceedings of the 24th International Conference on World Wide Web*. Geneva: International World Wide Web Conferences Steering Committee.

[B133] Tantardini M. Ieva F. Tajoli L. Piccardi C. (2019). Comparing methods for comparing networks. Sci. Rep. 9, 1. 10.1038/s41598-019-53708-y 31772246 PMC6879644

[B134] Tong F. (2019). What is Geometric Deep Learning? Available online at: https://flawnsontong.medium.com/what-is-geometric-deep-learning-b2adb662d91d#::text=In%20traditional%20Deep%20Learning%2C%20dimensionality,number%20of%20features%20it%20has (accessed June 29, 2023).

[B135] Torricelli M. Karsai M. Gauvin L. (2020). weg2vec: Event embedding for temporal networks. Sci. Rep. 10, 1. 10.1038/s41598-020-63221-2 32346033 PMC7189270

[B136] Townshend R. J. L. Eismann S. Watkins A. M. Rangan R. Karelina M. Das R.. (2021). Geometric deep learning of RNA structure. Science 373, 1047–1051. 10.1126/science.abe5650 34446608 PMC9829186

[B137] Tsitsulin A. Mottin D. Karras P. Bronstein A. Müller E. (2018). “NetLSD,” in Proceedings of the 24th ACM SIGKDD International Conference on Knowledge Discovery & *Data Mining*. New York: ACM.

[B138] Vaudaine R. Cazabet R. Largeron C. (2020). “Comparing the preservation of network properties by graph embeddings,” in Lecture Notes in Computer Science, M. R. Berthold, A. Feelders, and G. Krempl. Cham: Springer International Publishing, 522–534.

[B139] Veličković P. Cucurull G. Casanova A. Romero A. Liò P. Bengio Y. (2018). “Graph attention networks,” in International Conference on Learning Representations. Available online at: OpenReview.net

[B140] von Luxburg U. (2007). A tutorial on spectral clustering. Stat. Comput. 17, 395–416. 10.1007/s11222-007-9033-z

[B141] Wang B. Chen Y. Sheng J. He Z. (2022). Attributed graph embedding based on attention with cluster. Mathematics 10, 4563. 10.3390/math10234563

[B142] Wang C. Pan S. Long G. Zhu X. Jiang J. (2017). “MGAE,” in Proceedings of the 2017 ACM on Conference on Information and Knowledge Management. New York: ACM.

[B143] Wang C. Wang C. Wang Z. Ye X. Yu P. S. (2020). Edge2vec. ACM Trans. Knowl. Discov. Data 14, 1–24. 10.1145/3391298

[B144] Wang D. Cui P. Zhu W. (2016). “Structural deep network embedding,” in Proceedings of the 22nd ACM SIGKDD International Conference on Knowledge Discovery and Data Mining. New York: ACM.

[B145] Weininger D. (1988). SMILES, a chemical language and information system. 1. introduction to methodology and encoding rules. J. Chem. Inform. Comp. Sci. 28, 31–36. 10.1021/ci00057a005

[B146] Williams L. Z. J. Fawaz A. Glasser M. F. Edwards A. D. Robinson E. C. (2021). “Geometric deep learning of the human connectome project multimodal cortical parcellation,” in Machine Learning in Clinical Neuroimaging, A. Abdulkadir, S. M. Kia, M. Habes, V. Kumar, J. M. Rondina, C. Tax, et al. Cham: Springer International Publishing, 103–112.

[B147] Wills P. Meyer F. G. (2020). Metrics for graph comparison: a practitioner's guide. PLoS ONE 15, e0228728. 10.1371/journal.pone.0228728 32050004 PMC7015405

[B148] Wu Y. Besson P. Azcona E. A. Bandt S. K. Parrish T. B. Breiter H. C.. (2022). A multicohort geometric deep learning study of age dependent cortical and subcortical morphologic interactions for fluid intelligence prediction. Sci. Rep. 12, 1. 10.1038/s41598-022-22313-x 36273036 PMC9588039

[B149] Wu Y.-H. Huang Y.-A. Li J.-Q. You Z.-H. Hu P.-W. Hu L.. (2023). Knowledge graph embedding for profiling the interaction between transcription factors and their target genes. PLoS Comput. Biol. 19, e1011207. 10.1371/journal.pcbi.1011207 37339154 PMC10313080

[B150] Xia J. Lin H. Xu Y. Tan C. Wu L. Li S.. (2023). Gnn cleaner: Label cleaner for graph structured data. IEEE Trans. Know. Data Eng. 2023, 1–12. 10.1109/TKDE.2023.3288002

[B151] Xie L. Shen H. Feng D. (2022). Structural–temporal embedding of large-scale dynamic networks with parallel implementation. Comp. Electrical Eng. 100, 107835. 10.1016/j.compeleceng.2022.107835

[B152] Xiong Z. Wang D. Liu X. Zhong F. Wan X. Li X.. (2019). Pushing the boundaries of molecular representation for drug discovery with the graph attention mechanism. J. Med. Chem. 63, 8749–8760. 10.1021/acs.jmedchem.9b00959 31408336

[B153] Xu M. (2021). Understanding graph embedding methods and their applications. SIAM Review 63, 825–853. 10.1137/20M1386062

[B154] Yan C. Chang Z. (2019). Modularized tri-factor nonnegative matrix factorization for community detection enhancement. Physica A. 533, 122050. 10.1016/j.physa.2019.122050

[B155] Yang C. Sun M. Liu Z. Tu C. (2017). “Fast network embedding enhancement via high order proximity approximation,” in Proceedings of the Twenty-Sixth International Joint Conference on Artificial Intelligence. Cape Town: International Joint Conferences on Artificial Intelligence Organization.

[B156] Yang F. Fan K. Song D. Lin H. (2020). Graph-based prediction of protein-protein interactions with attributed signed graph embedding. BMC Bioinformat. 21, 1. 10.1186/s12859-020-03646-8 32693790 PMC7372763

[B157] Yang H. Pan S. Zhang P. Chen L. Lian D. Zhang C. (2018). “Binarized attributed network embedding,” in 2018 IEEE International Conference on Data Mining (ICDM). Singapore: IEEE, 1476–1481.

[B158] Yang M. Zhou M. Xiong H. King I. (2022a). Hyperbolic temporal network embedding. IEEE Trans. Knowl. Data Eng. 35, 11489–11502. 10.1109/TKDE.2022.3232398

[B159] Yang R. Shi J. Huang K. Xiao X. (2022b). “Scalable and effective bipartite network embedding,” in Proceedings of the 2022 International Conference on Management of Data. New York: ACM.

[B160] Ye W.-C. Wang J.-C. (2022). “Multilabel classification based on graph neural networks,” in Artificial Intelligence. London, UK: IntechOpen.

[B161] Ying R. You J. Morris C. Ren X. Hamilton W. L. Leskovec J. (2018). “Hierarchical graph representation learning with differentiable pooling,” in Proceedings of the 32nd International Conference on Neural Information Processing Systems, NIPS'18. Red Hook, NY, USA: Curran Associates Inc, 4805–4815.

[B162] Yu L. Cheng M. Qiu W. Xiao X. Lin W. (2022). idse-HE: Hybrid embedding graph neural network for drug side effects prediction. J. Biomed. Inform. 131, 104098. 10.1016/j.jbi.2022.104098 35636720

[B163] Yue X. Wang Z. Huang J. Parthasarathy S. Moosavinasab S. Huang Y.. (2019). Graph embedding on biomedical networks: methods, applications and evaluations. Bioinformatics 36, 1241–1251. 10.1093/bioinformatics/btz718 31584634 PMC7703771

[B164] Zhang D. Yin J. Zhu X. Zhang C. (2017). “User profile preserving social network embedding,” in Proceedings of the Twenty-Sixth International Joint Conference on Artificial Intelligence. Cape Town: International Joint Conferences on Artificial Intelligence Organization.

[B165] Zhang D. Yin J. Zhu X. Zhang C. (2020). Network representation learning: A survey. IEEE Trans. Big Data 6, 3–28. 10.1109/TBDATA.2018.2850013

[B166] Zhang H. Zha Z.-J. Yan S. Wang M. Chua T.-S. (2012). “Robust non-negative graph embedding: Towards noisy data, unreliable graphs, and noisy labels,” in 2012 IEEE Conference on Computer Vision and Pattern Recognition. Providence, RI: IEEE, 2464–2471.

[B167] Zhang S. Tong H. Xu J. Maciejewski R. (2019a). Graph convolutional networks: a comprehensive review. Computat. Soc. Netw. 6, 1. 10.1186/s40649-019-0069-y PMC10615927 37915858

[B168] Zhang X. He Y. Brugnone N. Perlmutter M. Hirn M. (2021a). Magnet: A neural network for directed graphs. Adv. Neural Inf. Process. Syst. 34, 27003–27015. 36046111 PMC9425115

[B169] Zhang X.-M. Liang L. Liu L. Tang M.-J. (2021b). Graph neural networks and their current applications in bioinformatics. Front. Genet. 12, 690049. 10.3389/fgene.2021.690049 34394185 PMC8360394

[B170] Zhang Y. Chen Q. Yang Z. Lin H. Lu Z. (2019b). BioWordVec, improving biomedical word embeddings with subword information and MeSH. Scient. Data 6, 1. 10.1038/s41597-019-0055-0 31076572 PMC6510737

[B171] Zhang Y.-J. Yang K.-C. Radicchi F. (2021c). Systematic comparison of graph embedding methods in practical tasks. Phys. Rev. E 104, 044315. 10.1103/PhysRevE.104.044315 34781460

[B172] Zhang Z. Cui P. Wang X. Pei J. Yao X. Zhu W. (2018). “Arbitrary-order proximity preserved network embedding,” in Proceedings of the 24th ACM SIGKDD International Conference on Knowledge Discovery & *Data Mining, KDD '18*. New York, NY, USA: Association for Computing Machinery, 2778–2786.

[B173] Zhao B.-W. Su X.-R. Hu P.-W. Ma Y.-P. Zhou X. Hu L. (2022). A geometric deep learning framework for drug repositioning over heterogeneous information networks. Brief. Bioinformatics 23, 6. 10.1093/bib/bbac384 36125202

[B174] Zhao F. Zhang Y. Lu J. (2021). ShortWalk: an approach to network embedding on directed graphs. Soc. Netw. Analy. Mining 11, 1. 10.1007/s13278-020-00714-y

[B175] Zhao S. Du Z. Chen J. Zhang Y. Tang J. Yu P. S. (2023). Hierarchical representation learning for attributed networks. IEEE Trans. Knowl. Data Eng. 35, 2641–2656.

[B176] Zheng S. Ma H. Wang J. Li J. (2018). “A computational bipartite graph-based drug repurposing method,” in Methods in Molecular Biology. Cham: Springer New York, 115–127. 10.1007/978-1-4939-8955-3_7 30547439

[B177] Zhou D. Hofmann T. Schölkopf B. (2004). “Semi-supervised learning on directed graphs,” in Advances in Neural Information Processing Systems, eds. L. Saul, Y. Weiss, and L. Bottou. New York: MIT Press.

[B178] Zhou D. Huang J. Schölkopf B. (2005). “Learning from labeled and unlabeled data on a directed graph,” in Proceedings of the 22nd international conference on Machine learning - *ICML*. New York: ACM Press.

[B179] Zhu Q. Jiang X. Zhu Q. Pan M. He T. (2019). Graph embedding deep learning guides microbial biomarkers' identification. Front. Genet. 10, 1182. 10.3389/fgene.2019.01182 31824573 PMC6883002

